# Comparison of Distal Radial, Proximal Radial, and Femoral Access in Patients with ST-Elevation Myocardial Infarction

**DOI:** 10.3390/jcm10153438

**Published:** 2021-08-02

**Authors:** Oh-Hyun Lee, Yongcheol Kim, Nak-Hoon Son, Ji Woong Roh, Eui Im, Deok-Kyu Cho, Donghoon Choi

**Affiliations:** 1 Division of Cardiology, Department of Internal Medicine, Yonsei University College of Medicine and Cardiovascular Center, Yongin Severance Hospital, Yongin 16995, Korea; decenthyun@yuhs.ac (O.-H.L.); nomgalda@yuhs.ac (J.W.R.); imeui97@yuhs.ac (E.I.); chodk123@yuhs.ac (D.-K.C.); cdhlyj@yuhs.ac (D.C.); 2 Data Science Team (Biostatistician), Center for Digital Health, Yongin Severance Hospital, Yonsei University College of Medicine, Yongin 16995, Korea; nhson@yuhs.ac

**Keywords:** radial artery, ST-elevation myocardial infarction, percutaneous coronary intervention, bleeding

## Abstract

Recent studies have indicated that distal radial access (DRA) is feasible in patients undergoing percutaneous coronary intervention (PCI). The present study aimed to compare DRA, proximal radial access (PRA), and femoral access (FA) in patients with ST-elevation myocardial infarction (STEMI) undergoing PCI. Data were analyzed for 109 patients with STEMI treated via primary PCI from March 2020 to May 2021. The success rate of DRA was 83.3% (35/42), including seven cases of failed puncture (puncture failure = 5, severe radial artery spasm = 2). Primary PCI via the DRA was successful in all 35 patients. After classifying the patients requiring crossover into a separate group, the percentage of the puncture time in the door-to-wiring time was 2.7% [2.2–4.3], 3.3% [2.3–4.0], 2.6% [1.2–4.9], and 27.0% [13.5–29.3] in the DRA (*n* = 35), PRA (*n* = 24), FA (*n* = 26), and crossover (*n* = 9) groups, respectively (*p* < 0.01). Only two local hematomas (≤5 cm) occurred in the DRA group, while one patient in the FA group required surgical treatment and a transfusion for an access-site vascular injury. When performed by an experienced operator, DRA may represent a feasible alternative to other access routes in select patients with STEMI undergoing PCI, such as those with a high risk of bleeding.

## 1. Introduction

Proximal radial access (PRA) for cardiac catheterization is associated with a better ability to achieve hemostasis, greater patient comfort, earlier ambulation, and shorter duration of hospitalization than femoral access (FA) [1]. Moreover, recent randomized trials and meta-analyses have demonstrated that PRA is associated with a reduced risk of access-site complications, major bleeding, and mortality in patients with acute coronary syndrome, when compared to FA [2,3,4,5]. Accordingly, current guidelines recommended PRA as the standard approach for percutaneous coronary intervention (PCI). In addition, for experienced radial operators, PRA is recommended over FA when performing primary PCI in patients with ST-elevation myocardial infarction (STEMI) [6,7].

Previous studies have demonstrated that the recently introduced strategy of distal radial access (DRA) is feasible and exhibits several advantages over PRA, including reductions in the risk of puncture-related injuries to the proximal radial artery and access-site complications such as significant bleeding [8,9,10,11,12]. More recently, a randomized trial demonstrated rates of radial artery occlusion are lower following DRA than following PRA [13]. From this perspective, DRA may represent an alternative access route for primary PCI in select patients with STEMI taking potent P2Y_12_ inhibitors (e.g., ticagrelor or prasugrel) or glycoprotein IIb/IIIa inhibitors [14]. However, there is a paucity of data regarding the comparison among vascular access routes in the setting of STEMI. Therefore, the present study aimed to compare the safety and feasibility of DRA, PRA, and FA in patients with STEMI undergoing PCI.

## 2. Materials and Methods

### 2.1. Patients and Study Design

From March 2020 to May 2021, patients who underwent primary PCI for STEMI at Yongin Severance Hospital were enrolled in the current study. A total of five experienced radial operators, defined as operators who perform at least 50% of all PCI procedures in patients with acute coronary syndrome via the radial approach, participated in this study [15]. Of these five operators, two were experienced DRA operators, and the other three had never utilized DRA at the beginning of patient registration in March 2020. The study protocol was approved by the Institutional Review Board of Yongin Severance Hospital (approval number: 9-2021-0069), which waived the requirement for informed consent owing to the retrospective observational study design.

### 2.2. Preparation for Each Vascular Access Route

Detailed information regarding DRA has been described previous studies of PCI via the DRA [12]. After procedure, hemostasis was achieved with a compressive bandage with gauze for 3 h in DRA [16]. During PRA, the arm was positioned by the side of the body on an arm board. For easier puncture, a soft roll was placed under the wrist to ensure hyperextension of the wrist. For left PRA, the hand was positioned over the left groin with a soft cushion kept under the left elbow, and the same positioning was used for left DRA. Following the PCI procedure, the radial sheath was removed, and a compression bandage was applied for 4 h. In the case of FA, ultrasound-guided puncture was performed in all patients to reduce the risk of access-site complications. After the procedure, the decision to use a vascular closure device (VCD), as well as the type of VCD, was left to the operator’s discretion.

### 2.3. Primary PCI Procedures

Immediately after the diagnosis of STEMI, all patients were given a loading dose of aspirin (300 mg), a P2Y_12_ receptor inhibitor (180 mg of ticagrelor or 300–600 mg of clopidogrel), and 5000 units of unfractionated heparin before the procedure if they were not previously taking these medications. The choice of medication was determined by each physician. Primary PCI was performed in accordance with the standard technique and current guidelines for STEMI [6]. Additional unfractionated heparin (50 to 70 U/kg) was administered during the procedure to maintain the activated clotting time at 250 to 300 s. Details regarding the treatment strategy including the sheath size, use of thrombus aspiration, use of glycoprotein IIb/IIIa inhibitors, intravascular imaging guidance, and the need for stent implantation were determined according to the physician’s discretion.

### 2.4. Definitions and Study Endpoints

STEMI was defined as new ST-segment elevation >0.1 mV in at least two contiguous leads and a new left bundle branch block on 12-lead electrocardiogram with a concomitant increase in cardiac markers (creatinine kinase-myocardial band [MB] or troponin T). Primary PCI was defined as PCI in patients presenting within 12 h of symptom onset or >12 h from symptom onset with clinical and/or electrocardiographic evidence of ongoing ischemia [6]. Successful vascular access was defined as the success of sheath cannulation. Puncture time was defined as the time interval from local anesthesia induction to successful sheath cannulation. Door-to-wiring-time (D2WT) was defined as the time elapsed from arrival of the patient at the emergency department to guide wire passage through the lesion. Major bleeding was defined as Bleeding Academic Research Consortium (BARC) type 3 or 5 bleeding [17].

The primary endpoint was the rate of access-site complications including major bleeding requiring transfusion or surgery, hematoma, and arterial occlusion. Secondary endpoints included the puncture success rate, success rate of primary PCI, and the percentage of puncture time in D2WT.

### 2.5. Statistical Analysis

Normally distributed continuous variables are expressed as means ± standard deviations (SD), while non-normally distributed continuous variables are expressed as medians and interquartile ranges (IQR). All categorical variables are presented as numbers with percentile values. Continuous variables were compared using one-way analyses of variance (ANOVAs), Kruskal-Wallis tests, Student’s *t*-tests, or Mann-Whitney *U*-tests, as indicated. Categorical variables were compared using chi-square tests. A two-sided *p* value of less than 0.05 was considered statistically significant. All statistical analyses were performed using SPSS statistical software (SPSS version 25.0 for Windows; IBM Corp., Armonk, NY, USA).

## 3. Results

**Figure 1:**
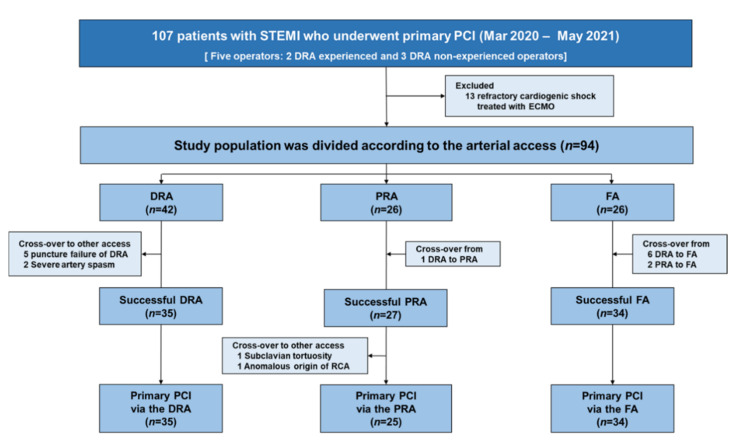
Study flowchart. Abbreviations: ECMO, extracorporeal membrane oxygenation; DRA, distal radial access; FA, femoral access; PCI, percutaneous coronary intervention; PRA, proximal radial access; RCA, right coronary artery; STEMI, ST-elevation myocardial infarction.

From March 2020 to May 2021, we identified 109 consecutive patients who underwent primary PCI for STEMI. Among them, 13 patients with refractory cardiogenic shock requiring extracorporeal membrane oxygenation were excluded, and a total of 97 patients were enrolled. DRA, PRA, and FA were firstly attempted in 42, 26, and 26 patients during the study period, respectively (Figure 1). The success rate of DRA was 83.3% (35/42). Among the seven cases of failed DRA, distal radial artery puncture failed in five patients, while sheath cannulation failed after successful puncture in two cases due to severe arterial spasms accompanied by pain. Crossover to PRA and FA was achieved in one and six patients, respectively. However, the success rate of primary PCI was 100% in all 35 patients in whom successful DRA was achieved (Table 1). Vascular access was ultimately successful in all cases of PRA when including crossover cases (initial PRA = 26, crossover from DRA to PRA = 1). However, due to subclavian tortuosity and anomalous origin of the right coronary artery (RCA), subsequent crossover to FA was required in two patients. Finally, 35, 25, and 34 patients were enrolled in the DRA, PRA, and FA groups for analysis, respectively. 

Baseline characteristics of the study population are presented in Supplementary Table S1. The overall average age of the patients was 60.4 ± 13.3 years (range: 29–88 years), and 86.2% (81/94) were male. The baseline characteristics did not significantly differ among the three groups, except that the hemoglobin level was lower in the FA group than in the other two groups.

Angiographic and procedural characteristics are listed in Supplementary Table S2. Temporary pacemaker implantation was performed more frequently in the FA group than in the DRA and PRA groups (*p* < 0.01). Total procedure time and contrast volume were comparable among the three groups. Primary PCI with a 6-French guiding catheter was performed in 77.1%, 100%, and 85.3% of patients in the DRA, PRA, and FA groups, respectively. Infarct-related artery, disease extent, total number of implanted stents, multivessel PCI, intravascular imaging-guided PCI, and the use of thrombus aspiration and glycoprotein IIb/IIIa inhibitors were comparable among the three groups. In the FA group, VCD was used in 91.2% (31/34) of patients.

**Figure 2:**
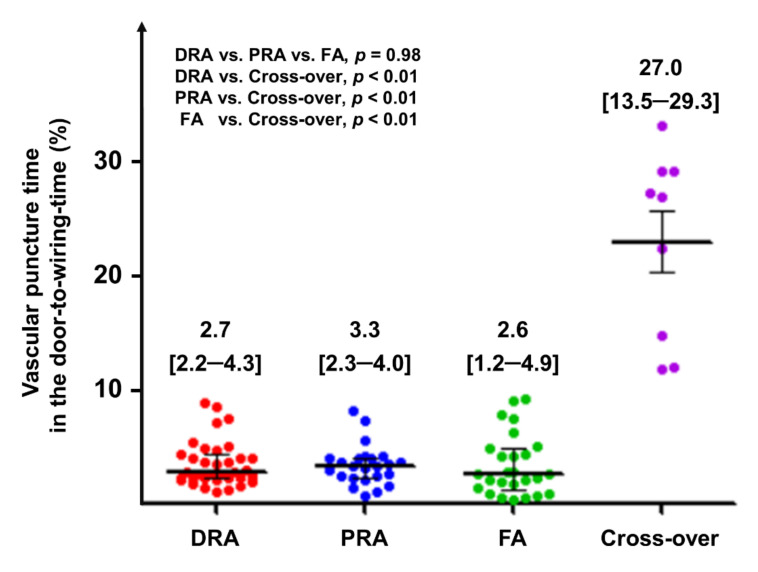
Percentage of the puncture time in the door-to-wiring time. Horizontal lines represent the median with the interquartile range. Abbreviations: DRA, distal radial access; FA, femoral access; PRA, proximal radial access.

After classifying patients requiring crossover of vascular access separately, mean puncture times in the DRA, RA, FA, and crossover groups were 116 ± 56, 101 ± 46, 129 ± 106, and 1033 ± 693 s, respectively (*p* = 0.03). To determine the safety of puncture for primary PCI, we also evaluated the percentage of puncture time in the D2WT, which was 2.7% [2.2–4.3], 3.3% [2.3–4.0], 2.6% [1.2–4.9], and 27.0% [13.5–29.3] in the DRA, RA, FA, and crossover groups, respectively (*p* < 0.01) (Table 2 and Figure 2).

Access-site complications are summarized in Table 3. Local hematoma less than 5 cm in diameter occurred in two (5.7%) patients in the DRA group, two (8.0%) patients in the PRA group, and four (11.8%) patients in the FA group (*p* = 0.66). One patient who underwent primary PCI via FA underwent subsequent surgery and a blood transfusion for damage to the femoral artery and vein during vascular access. 

## 4. Discussion

To the best of our knowledge, this is the first clinical study to compare the safety and feasibility of DRA, PRA, and FA for primary PCI in patients with STEMI. The principal findings were as follows: (1) The success rate of DRA was 83.3% (35/42), including five cases of failed puncture and two cases of failed sheath cannulation. (2) Primary PCI was successful in all 35 patients with successful DRA. (3) Rates of access-site complications for DRA were low and comparable to those for PRA and FA, and no major bleeding complications were observed in the DRA group. 

Several studies have reported that the success rates of DRA for coronary angiography (CAG) or PCI range from 88.0–95.5% [9,10,11]. A recent study further reported a DRA success rate of 92.8% in patients with STEMI [14]. However, the success rate of DRA for primary PCI was relatively lower in our study at 83.3% (35/42). Detailed information for the patients in whom DRA failure was observed is summarized in Supplementary Table S3. The lower success rate in the present study may be explained by our inclusion of three inexperienced DRA operators who may need to overcome a learning curve for DRA. Recently, we reported that 200 cases would be needed to overcome the learning curve with a consistently high success rate (>94.0%), and that female sex and systolic blood pressure (SBP) <120 mm Hg are predictors of failed DRA [18]. Therefore, DRA should be considered in select patients with STEMI (male patients and those with high SBP) undergoing primary PCI when the operator has utilized DRA in over 200 cases of CAG or PCI. 

In the current study, the proportion of puncture time in D2WT was also comparable among the DRA, PRA, and FA groups when crossover cases were excluded. In addition, the proportion of puncture time within 3 and 5 min was very high for DRA (88.6% [31/35] and 100% [35/35] patients, respectively). Previous studies have reported puncture times of 1.19 to 3.9 min for DRA [11,14,19,20]. Thus, the puncture time of DRA in patients with STEMI in the current study is acceptable when compared with puncture times for other vascular access routes.

There has been some concern regarding the possibility of delays in puncture time, which may result in a delay in D2WT in patients presenting with STEMI because the distal radial artery is smaller than the proximal radial artery or femoral artery [10]. Although puncture time was longer for DRA than for PRA among patients with successful vascular access, our analysis of real-world data indicated that this difference was not statistically significant and did not lead to a delay in D2WT. In contrast, among patients with initial puncture failure requiring conversion to another site, we observed significant increases in puncture time and the proportion of puncture time to D2WT. Therefore, as suggested in our previous study, operators should remain aware that delayed puncture over 5 min can be considered an indication of the need to change the puncture site for the safety of patients [14].

In the present study, successful primary PCI was achieved in all 35 DRA cases, and left DRA was performed in 34 (97.1%) patients in DRA group. Although right PRA is often used for operator comfort, it has the disadvantage of potential anatomic variations and poor back-up force due to the S-shaped geometry of the subclavian–innominate–aorta axis [21]. Furthermore, the risk of embolic stroke is increased because the catheter needs to be passed from the innominate artery into the ascending aorta where the right carotid artery comes off [22]. In contrast, the left PRA is advantageous due to the ease with which the catheter can be manipulated (similar to FA); however, physical discomfort remains a barrier for some operators. The left DRA can overcome this barrier, as the patient’s elbow is slightly bent, and the left hand is positioned above the left groin. In addition to these strengths, a previous study reported a 100% success rate of PCI via the left DRA for bifurcation lesions without crossover [12]. Further large-scale randomized trials should be conducted to compare the feasibility of left and right DRA for complex PCI including primary PCI in patients with STEMI.

Previous studies have reported no major complications (including major bleeding) in patients undergoing CAG or PCI via the DRA [9,10,11,12,14,20,23,24]. In the present study, we observed no major bleeding in the DRA group, for which rates of P2Y_12_ inhibitor and glycoprotein IIb/IIIa inhibitor use were 83% and 26%, respectively. Local hematoma <5 cm in diameter was observed in only two patients, although both fully recovered within 1 month. In contrast, major bleeding requiring transfusion and surgical treatment due to access-site vascular injury occurred in patients with FA. Therefore, DRA may represent an alternative access route in patients with a high risk of bleeding and in those in whom bleeding complications must be minimized. 

This study had some limitations. First, the study was a retrospective, observational study—a design associated with inherent selection and information bias. Second, the small number of patients included in the study limits its statistical power. Third, puncture failure may still be a barrier to the success of DRA. Further study is needed on whether ultrasound-guided puncture can reduce puncture failure. Fourth, since ultrasound guidance was used in all cases of FA, this may have resulted in research bias, especially with regard to puncture time. Fifth, the lack of routine postprocedural ultrasonography for the puncture site may have contributed to underestimation of puncture-site complications. Despite these limitations, our findings are expected to aid interventional cardiologists in understanding the feasibility of DRA for primary PCI in the setting of STEMI.

## 5. Conclusions

Despite the relatively low success rate of DRA in patients with STEMI, the success rate of primary PCI was 100% in cases of successful DRA. Moreover, there were no major access-site complications in patients with successful DRA, and puncture time and the proportion of puncture time in the D2WT were comparable to those for PRA and FA. For highly experienced operators who have overcome the learning curve, primary PCI via the DRA (especially on the left side) may represent a feasible alternative in select patients with STEMI, such as those with a high risk of bleeding.

## Figures and Tables

**Table 1:** Detailed information regarding vascular access routes.

Characteristics	DRA	PRA	FA
Attempted puncture (number of patients)	42	27	34
Puncture success rate	35 (83.3)	27 (100)	34 (100)
Crossover to other access site	7	2	0
Proximal radial access	1 (14.3)	0	0
Femoral access	6 (85.7)	2 (100)	0
Patients with successful access	35	27	34
Left-sided access	34 (97.1)	11 (40.7)	2 (7.7)
Success rate of diagnostic CAG	35 (100)	25 (92.6)	34 (100)
Success rate of primary PCI	35 (100)	25 (92.6)	34 (100)

Data are presented as number (%). Abbreviations: DRA, distal radial access; PRA, proximal radial access; FA, femoral access; CAG, coronary angiography; PCI, percutaneous coronary intervention.

**Table 2:** Detailed information regarding vascular access routes after separating crossover cases.

Characteristics	DRA Group (*n* = 35)	PRA Group (*n* = 24)	FA Group (*n* = 26)	Crossover Group (*n* = 9)	*p* Value
Puncture time, s					<0.01 ^†^
Mean ± standard deviation	116.1 ± 56.1	100.8 ± 46.0	129.2 ± 105.9	1033.3 ± 692.5	
Median (IQR)	91 (78–150)	87 (61–120)	120 (53–195)	960 (390–1380)	
Puncture time <3 min	31 (88.6)	22 (91.7)	20 (76.9)	0	<0.01
Puncture time <5 min	35 (100)	24 (100)	25 (96.2)	1 (11.1)	<0.01
Door-to-wiring time (D2WT), min	62.7 ± 28.5	59.2 ± 37.8	80.1 ± 51.8	69.4 ± 34.5	0.17
Proportion of puncture time to D2WT, %	3.5 ± 2.0	3.4 ± 1.8	3.4 ± 2.7	23.1 ± 8.2	<0.01 ^§^

Data are presented as mean ± SD, number (%), or median (quartile 1–quartile 3). † *p* value for DRA vs. PRA, 0.27; DRA vs. FA, 0.54; PRA vs. FA, 0.23; DRA vs. crossover, <0.01; PRA vs. crossover, <0.01, FA vs. crossover, <0.01. § *p* value for DRA vs. PRA, 0.84; DRA vs. FA, 0.89; PRA vs. FA, 0.98; DRA vs. crossover, <0.01, PRA vs. crossover, <0.01, FA vs. crossover, <0.01. Abbreviations: IQR, interquartile range; DRA, distal radial access; PRA, proximal radial access; FA, femoral access.

**Table 3:** Outcomes.

Characteristics	DRA (*n* = 35)	PRA (*n* = 25)	FA (*n* = 34)	*p* Value
Access-site complications	2 (5.7)	2 (8.0)	4 (11.8)	0.66
Local hematoma (≤5 cm)	2 (5.7)	2 (8.0)	4 (11.8)	0.66
Local hematoma (>5 cm)	0	0	0	-
Bleeding requiring transfusion or surgery	0	0	1 (2.9)	0.41
Puncture site injury	0	0	1 (2.9)	0.41
Pseudoaneurysm	0	0	0	-
Arteriovenous fistula	0	0	0	-
Occlusion	0	0	0	-
Hemorrhagic stroke	0	0	0	-
Myocardial infarction	0	0	0	-
BARC bleeding	0	0	1 (2.9)	
Type 3a	0	0	0	0.41
Type 3b	0	0	1 (2.9)	
Type 5	0	0	0	

Data are presented as mean ± SD or number (%). Abbreviation: DRA, distal radial access; PRA, proximal radial access; FA, femoral access; BARC, Bleeding Academic Research Consortium.

## Data Availability

The underlying data set is available from the corresponding author upon reasonable request.

## Supplementary Materials

The following are available online at https://www.mdpi.com/article/10.3390/jcm10153438/s1, Table S1: Baseline characteristics; Table S2: Angiographic and procedural characteristics; Table S3: Characteristics of patients with failed DRA.

## Clinical Trial Registration

https://www.clinicaltrials.gov/ct2/show/NCT04941638 (28 June 2021).
