# Supplementary material for: Comparison of Distal Radial, Proximal Radial, and Femoral Access in Patients with ST-Elevation Myocardial Infarction
Source: J Clin Med. 2021 Aug 2;10(15):3438. doi: 10.3390/jcm10153438 (PMC8348546; doi:10.3390/jcm10153438)
Supplement: Supplementary file 1 [file jcm-10-03438-s001.zip › jcm-1304523-supplementary.pdf]

Supplementary Table S1. Baseline characteristics.

Characteristics	DRA (n=35)	PRA (n=25)	FA (n=34)	*p* value
Age, years	60.3 ± 14.3	59.0 ±13.1	61.5 ± 12.6	0.78
Age ≥75 years	7 (20.0)	4 (16.0)	6 (17.6)	0.92
Male	30 (85.7)	24 (96.0)	27 (79.4)	0.19
Body mass index, kg/m^2	24.5 ± 3.2	26.2 ±2.5	25.2 ± 5.1	0.23
**Vital signs**				
Systolic BP, mmHg	127.9 ± 20.3	127.6 ± 25.3	128.7 ± 23.4	0.98
Diastolic BP, mmHg	76.0 ± 12.7	77.1 ± 13.3	70.9 ± 11.8	0.12
Heart rate, beats per min	74.9 ± 15.4	77.5 ± 20.7	76.4 ± 15.3	0.84
**Cardiovascular risk factors**				
Hypertension	13 (37.1)	10 (40.0)	16 (47.1)	0.69
Diabetes mellitus	8 (22.9)	6 (24.0)	12 (35.3)	0.46
Current smoker	15 (42.9)	12 (48.0)	13 (38.2)	0.75
Dyslipidemia	8 (22.9)	11 (44.0)	14 (41.2)	0.16
Atrial fibrillation	2 (5.7)	4 (16.0)	3 (8.8)	0.40
CKD ≥ stage 3	1 (2.9)	3 (12.0)	5 (14.7)	0.22
Prior stroke	0	0	1 (2.9)	0.41

Prior MI	0	2 (8.0)	2 (5.9)	0.27
Prior PCI	2 (5.7)	4 (16.0)	3 (8.8)	0.40
Prior bypass surgery	0	0	0	-
**Killip class 3/4**	8 (22.9)	3 (12.0)	12(35.3)	0.28
**LVEF, %**	47.9 ± 10.8	46.6 ± 9.9	47.3 ± 10.9	0.90
**Laboratory findings**				
Hemoglobin, g/dL	15.3 ±1.5	15.2 ± 1.4	14.3 ± 1.8	0.03
Platelet count, $10^3/\mu L$	236.2 ± 60.3	254.1 ± 90.1	262.1 ± 64.3	0.30
Prothrombin time, INR	0.98 ± 0.05	0.99 ± 0.04	1.02 ± 0.11	0.06
Creatinine, mg/dL	0.90 ± 0.28	0.97 ± 0.35	1.18 ± 1.55	0.45
eGFR, ml/min/1.73 m^2	82.5 ± 13.9	78.4 ± 15.8	75.7 ± 20.9	0.26
Peak CK-MB, ng/ml	144.5 ± 169.4	82.0 ± 130.0	173.8 ± 166.7	0.09
Peak troponin T, pg/ml	12110.3 ± 22596.6	6109.3 ± 3829.9	5110.2 ± 5653.9	0.10
**$P2Y_{12}$ inhibitor before PCI**				0.15
Clopidogrel	6 (17.1)	1 (4.0)	2 (5.9)	
Ticagrelor	29 (82.9)	24 (96.0)	32 (94.1)	
Prasugrel	0	0	0	
**Medications at discharge**				
Aspirin	35 (100)	25 (100.0)	34 (100)	-

$P2Y_{12}$ inhibitor				
Clopidogrel	6 (17.1)	5 (20.0)	7 (20.6)	0.93
Ticagrelor	27 (77.1)	20 (80.0)	26 (76.5)	0.95
Prasugrel	2 (5.7)	0	1 (2.9)	0.46
Warfarin	2 (5.7)	0	0	0.18
DOAC	1 (2.9)	4 (16.0)	3 (8.8)	0.20
ACEi or ARB	12 (34.3)	11 (44.0)	20 (58.8)	0.12
Beta-blocker	21 (60.0)	20 (80.0)	20 (58.8)	0.18
Statin	35 (100)	25 (100)	34 (100)	-

Data are presented as the mean ± SD or number (%).

Abbreviations: DRA, distal radial access; PRA, proximal radial access; FA, femoral access; BP, blood pressure; CKD, chronic kidney disease; MI, myocardial infarction; PCI, percutaneous coronary intervention; LVEF, left ventricular ejection fraction; INR, international normalized ratio; eGFR, estimated glomerular filtration rate; CK-MB, creatinine kinase myocardial band; DOAC, direct oral anticoagulants; ACEi, angiotensin converting enzyme inhibitor; ARB, angiotensin receptor blocker.

Supplementary Table S2. Angiographic and procedural characteristics.

Characteristics	DRA (n=35)	PRA (n=25)	FA (n=34)	*p* value
Infarct-related artery				0.38
LM	0	0	1 (2.9)	
LAD	19 (54.3)	12 (48.0)	15 (44.1)	
LCX	4 (11.4)	1 (4.0)	5 (14.7)	
RCA	10 (28.6)	12 (48.0)	13 (38.2)	
Ramus intermedius	2 (5.7)	0	0	
CAD extent				0.11
1VD	16 (45.7)	12 (48.0)	14 (41.2)	
2VD	16 (45.7)	6 (24.0)	9 (26.5)	
3VD	1 (2.9)	4 (16.0)	9 (26.5)	
LM involvement	2 (5.7)	3 (12.0)	2 (5.9)	
Lesion type, B2/C	35 (100)	25 (100)	34 (100)	-
PCI				
With stenting	32 (91.4)	24 (96.0)	31 (91.2)	0.77
Imaging-guided PCI				
IVUS	8 (22.9)	4 (16.0)	6 (17.6)	0.28
OCT	7 (20.0)	6 (24.0)	2 (5.9)	

Multi-vessel PCI	1 (2.9)	0	2 (5.9)	0.44
Total number of implanted stents	1.1 ±0.5	1.2 ±0.6	1.3 ± 0.8	0.21
Implantation of ≥2 stents	5 (14.3)	5 (20.0)	10 (29.4)	0.30
Thrombus aspiration	19 (54.3)	8 (32.0)	17 (50.0)	0.21
Temporary pacemaker	0 (0)	1 (4.0)	8 (23.5)	<0.01
Glycoprotein IIb/IIIa inhibitor use	9 (25.7)	8 (32.0)	14 (41.2)	0.39
Guiding catheter				
6-Fr	27 (77.1)	25 (100)	29 (85.3)	
7-Fr	7 (20.0)	0	5 (14.7)	<0.01
Sheathless	1 (2.9)	0	0	
Guiding catheter				
EBU or Amplatz type	28 (80.0)	15 (60.0)	24 (70.6)	0.24
Judkins type	7 (20.0)	10 (40.0)	10 (29.4)	
Vascular closure device use			31 (91.2)	
Perclose			12 (35.3)	
Femoseal			18 (52.9)	
Mynx grip			1 (2.9)	
Manual compression			3 (8.8)	

Total procedure time, min	50.2 ± 17.6	43.6 ± 26.1	55.0 ± 27.4	0.20
Total contrast volume, cc	234.0 ± 58.6	216.8 ± 92.5	258.2 ± 97.3	0.17

Data are presented as mean ± SD or number (%).

Abbreviations: DRA, distal radial access; PRA, proximal radial access; FA, femoral access; LM, left main artery; LAD, left anterior descending artery; LCX, left circumflex artery; RCA, right coronary artery; CAD, coronary artery disease; PCI, percutaneous coronary intervention; IVUS, intravascular ultrasound; OCT, optical coherence tomography.

Supplementary Table S3. Characteristics of patients with failed DRA.

No.	Sex	Age	Systolic BP, mmHg	Cross-over	Distal Radial Operator
1	Female	46	162	Proximal radial	Non-experienced
2	Female	47	134	Femoral	Non-experienced
3	Male	40	91	Femoral	Experienced
4	Female	58	114	Femora	Non-Experienced
5	Male	61	108	Femoral	Non-Experienced
6	Male	50	98	Femoral	Experienced
7	Female	44	111	Femoral	Non-Experienced

Abbreviations: DRA, distal radial access; BP, blood pressure.
